# G691S/S904S polymorphism in the *RET* protooncogene of a 25-year-old medical student with bilateral pheochromocytoma

**DOI:** 10.4103/0971-6866.50868

**Published:** 2009

**Authors:** Borros Arneth

**Affiliations:** Institute of Clinical Chemistry and Laboratory Medicine, University Mainz, Mainz, Germany

**Keywords:** Genetics, multiple endocrine neoplasia, pheochromocytoma

## Abstract

The case of a 25-year-old medical student with bilateral pheochromocytoma is described. Following diagnostic testing, tumors were surgically removed. Genetic analysis revealed that the patient is a heterozygote with the following mutations on opposite homologs: G691S (exon 11) and S904S (TCC-TCG, exon 15), suggesting the diagnosis of multiple endocrine neoplasia 2A (MEN2A). A diagnosis of MEN2 would be an indication of thyroidectomy in this patient. Although this mutation is described in the literature, it has no known connection to pheochromocytomas. Therefore, it is unknown whether there is a causal connection between the G691S genotype and the pheochromocytomas in this patient. If so, G691S is to be added to the list of genotypes causing MEN2A. Here, the procedure of sequencing the RET protooncogene is described and a possible association between the G691S genotype and MEN2A is discussed.

## Introduction

A Mutations on opposite homologs: G691S (exon 11) and S904S (TCC-TCG, exon 15), have been described in the literature, however, it has no known connection to pheochromocytomas. The case of a 25-year-old patient with bilateral pheochromocytoma with this mutation is described. The procedure of sequencing the RET protooncogene is described and a possible association between the G691S genotype and MEN2A is discussed.

## Case Report

A 25-year-old medical student attending a lecture on pheochromocytoma and its symptoms recognized the symptoms from his personal experience, but he had not previously attributed his symptoms to an abnormal condition. In recent years, his resting heart rate had risen from 60 to 80 beats/min and his blood pressure has risen from 120/60 to 140-150/80-90 mmHg. Although his blood pressure and heart rate usually remained at the upper end of the normal range, both increased with minimal exertion. He experienced nausea, dizziness, and pallor when his intra-abdominal pressure was increased by strong physical activity, but he remained unconcerned about his symptoms.

The source of his symptoms was revealed approximately 15 months later, when the young medical student volunteered to participate as a normal subject in a demonstration of abdominal ultrasound as part of the student curriculum. Instead of demonstrating a normal abdomen, his ultrasound revealed a 6-cm tumor dorsal to the liver at the upper pole of the right kidney. Before the ultrasound, no one had suspected the diagnosis of pheochromocytoma in this patient.

The patient was admitted for testing, which established the diagnosis of bilateral pheochromocytoma.

Laboratory tests revealed the following: adrenaline in 24-h urine was 58–108 µg/day (normal range < 10 µg/day) and normetanephrine in 24-h urine was 1305 µg/day (normal range, 30–440 µg/day). The clonidine suppression test revealed elevated basal adrenaline, which remained elevated after administration of Clonidine (failure of suppression).

Long-term (LT) electrocardiogram showed sinus rhythm with a heart rate of up to 137 beats/min. Electrocardiogram showed preterminal-negative T waves in lead III. LT Riva-Rocci (RR) was evaluated together with the LT electrocardiogram. Blood pressure measurement revealed that 40% of the systolic pressures were greater than 135 mmHg and 21% of the diastolic values were greater than 85 mmHg. Echocardiography revealed left ventriclar hypertrophy, with preserved systolic function and impaired diastolic relaxation as well as minimal tricuspidal valve insufficiency. MIBG, iodine-131-meta-iodobenzylguanidine scintigraphy showed inhomogeneous nuclide uptake in the liver and in both the adrenals.

Confirming the diagnosis, retroperitoneal computed tomography (CT) with and without contrast revealed a right-sided tumor with a diameter of 6 cm and a left-sided tumor with a diameter of approximately 3 cm. By iodine-131-meta-iodobenzylguanidine (MIBG), the left-sided tumor showed enhanced nuclide uptake. CT of the thorax with IV contrast showed a normal thorax; however, a 1.6 cm region of enhancement was seen in liver segment IVa. Further investigations followed to exclude metastatic pheochromocytoma.

CT of the liver with and without contrast as well as a CT of the abdomen with oral contrast showed a 1.5 cm hyperarterialised diffuse lesion in segment IVa of the liver, appearing with a diameter of 2.0 cm in the venous phase. The spleen was large but otherwise unremarkable; however, a 1.5 cm accessory spleen was noted. Both pheochromocytomas were visualized, measuring 6.1 × 6.0 cm on the right and 2.8 × 2.3 cm on the left.

Radionuclide examination of the mesenteric lymph nodes was performed using 100 MBq 111-indium-octreotide followed by a SPECT OPS 3-724. Low-grade expression of somatostatin receptors was found for both pheochromocytomas.

Abdominal magnetic resonance tomography with and without contrast revealed a lesion highly suspicious for metastasis in the left liver. Positron emission tomography with an 18-F-Dopa and an 18-F-FDG showed multifocal enrichment of both adrenals and a bland liver.

Bilateral adrenalectomy was performed, with resection of the surrounding retroperitoneal fatty tissue and the superior portion of segment IV of the liver. Postoperatively, the patient was treated with hydrocortisone 30 mg/day and astonine H 0.1 mg/day, which were administered for prevention of an Addisonian crisis. The surgical biopsy of the liver segment IV showed focal nodular hyperplasia.

Because the patient presented with bilateral pheochromocytomas, DNA sequencing for multiple endocrine neoplasia 2 (MEN2) screening was performed. The patient was treated by an endocrinologist for diffuse goiter, with weekly iodine. The therapeutic regimen was changed to Cortisone Ciba 37.5 mg/day. Astonin^®^ H was discontinued because the blood pressure remained stable without this medicine also. Once the patient was clinically stable, genetic investigation continued. To exclude in this patient, exons 10, 11, 12, 13, 14, 15, and 16 of the *RET* oncogene (RET on chromosome 10, 10q11.2) were sequenced. None of the common MEN2 mutations were found; however, the heterozygote genotype G691S/S904S was revealed. To date, this genotype has not been described in MEN2A; however, this genotype has been described in patients with C-cell carcinoma of the thyroid, raising the question of causality.

DNA extraction was carried out with the High Pure^®^ polymerase chain reaction (PCR) template preparation kit from Roche (Roche Diagnostics, Indianapolis, Indiana, USA).

Genomic DNA was stored at -20°C (-4°F).

PCR of the *RET* protooncogene exons was carried out with primers listed in Table 1 according to the protocol in Table 2.

**Table 1:** Primer

Exon	Primer sequence	Name	Tm	PCR product(bp)
10	GGACACTGCCCTGGAAATAT	ex10 F	55,0	275
	TGTTGGGACCTCAGATGTGCTG	ex10 R	55,3	
11	ATACGCAGCCTGTACCCAGT	ex11 F	57,2	497
	CACAGACTGTCCCCACACAG	ex11 R	57,1	
12	CCCCTGTCATCCTCACACTT	ex 12 F	56,4	300
	CTCTTCAGGGTCCCATGCT	ex 12 R	56,6	
13	TGACCTGGTATGGTCATGGA	ex 13 F	54,6	249
	GGAGAACAGGGCTGTATGGA	ex 13 R	55,9	
14	AAG ACC CAA GCT GCC TGA C	ex 14 F	57,6	350
	CAT GGT GGG CTA GAG TGT GG	ex 14 R	58,1	
15	CTGGTCACACCAGGCTGAG	ex 15 F	60,5	288
	CGGTATCTTTCCTAGGCTTCC	ex 15 R	60,5	
16	TCTCCTTTACCCCTCCTTCC	ex 16 F	55,8	176
	TGTAACCTCCACCCCAAGAG	ex 16 R	56,6	

**Table 2:** Polymerase chain reaction

11 μl	H_2_O
1 μl	MgCl_2_
2 μl	Forward primer
2 μl	Reverse primer
2 μl	Master mix
2 μl	DNA
20 μl	Total volume

For the temperature protocol, see Table 3. PCRs are optimized for the Light Cycler^®^ (Roche).

**Table 3:** Temperature protocol

Temperature (°C)	Time (s)	For exon	Cycles
96	600		
96	10		*
55	5	10,12,13	*
60	5	11,14,15,16	*
72	12		*
*35 cycles			
40	60		

For PCR product purification, a Qiaquick^®^ PCR purification kit from Qiagen (Qiagen, Valencia, California, USA) was used.

Cycle Sequencing was carried out with the Dye Terminator Sequenching Kit^®^ from Beckman (Beckman Coulter, Fullerton, California, USA) on a GenAmp 2400 thermocycler from Perkin Elmer (Perkin Elmer Waltham, Massachusetts, USA). The protocol is described in Table 4 and the temperature protocol is described in Table 5.

**Table 4:** Cycle sequencing

8 μl	H_2_O
8 μl	Master mix
2 μl	Forward or reverse primer
2 μl	PCR product

**Table 5:** Temperature protocol cycle sequencing

Temperature (°C)	Time	Cycles
96	20 s	*
50	20 s	*
60	4 min	*
*30 cycles		
4		

Dye terminators are eliminated using Centrisep^®^ columns from EMP-Biotech (EMP-Biotech, Berlin, Germany).

Dye-labeled DNA fragments are separated with a CEQ 2000 Sequencer Beckman Coulter, based on capillary electrophoresis after denaturation of the DNA fragments by adding Formamid (35 µl SLS) according to the manufacturer′s instructions.

Sequencher^®^ software is used for the analysis of the sequences.

The PCR amplifacation products can be sequenced easily with the procedure described.

If a mutation is found in one strand, it is confirmed by sequencing of the opposite strand. If a mutation of the *RET* protooncogene is confirmed by sequencing both strands, the result is communicated to the clinician. If a mutation is found, close relatives of the proband should be offered sequencing of the relevant exon. In individuals ascertained as relatives of an affected individual, it is not necessary to sequence exons other than the one containing the mutation in the index case.

## Discussion

Interestingly, it was recently shown that probably also within the case of the scientific first-description of pheochromocytomas, a MEN disease was the basis.[1] It concerned a bilateral tumor also in that case, as it is here.[1]

In the patient described in this study, gene sequencing demonstrated the heterozygote genotype G691S (exon 11) and S904S (TCC-TCG, exon 15) on opposite homologues of chromosome 10. The occurrence of bilateral pheochromocytomas in this patient suggests the diagnosis of MEN 2A or a mild 2B. Because patients with MEN2 are at high risk for medullary carcinoma of the thyroid, accurate diagnosis is critical. If a diagnosis of MEN2 is established, prophylactic thyroidectomy would be recommended.

Although this mutation has been described in the literature, no causal connection has been established with pheochromocytoma. Robledo![2] and Fernandez[3] refer to the G691S/S904S genotype as a “genetic modifier” while Lesueur[4] and Elisei[5] report a higher frequency of the polymorphism in patients with medullary carcinoma. The question of a causal connection between the G276S genotype and the pheochremocytoma remains unresolved. If a connection is established, G691S/S904S would be added to the list of genotypes causing MEN2.
